# Notes and News

**Published:** 1894-06-02

**Authors:** 


### Notes and News.

Collected and Communicated.

MEETINGS OF THE WEEK.

Royal Hospital for Incurables, Putney, Festival Dinner, Hotel Metropole, Thursday, May 31st.

St. Mary's Hospital, Festival Dinner, Hotel Metropole, Friday, June 1st.

North London Hospital for Consumption, Festival Dinner, Hotel Metropole, Saturday, June 2nd.

City of London Hospital for Diseases of Chest, Festival Dinner, Holborn Restaurant, Monday, June 4th.

Hospital for Women, Soho Square, Annual General Meeting, Thursday, June 7th, 3 p.m.

Hospital Sunday falls on June 10th this year. The Lord Mayor and Sheriffs will attend the services at St. Paul's Cathedral in the morning, and that of Westminster Abbey in the afternoon.

Small-pox has not yet disappeared from Chicago. The pest house is crowded, and the disease has appeared at the county hospital. There is, however, little appearance of general anxiety, and no schools have been closed, nor have any sanitary regulations been enforced.

In a recent number we drew attention to Dr. Collingridge's report on the health of sailors, and pointed out the prevalence of phthisis amongst them. This, of course, applied to English sailors. Since then a perusal of the report on the Japanese navy has revealed a like tendency on the part of Japanese sailors to diseases of the respiratory organs. Next to fevers, to which oriental navigators are especially subjected, the fatality is greatest in the direction we have pointed out.

A prominent figure has passed away from the world of science in the person of Mr. Georges Romanes. He was associated with all the theories and discussions on natural selection and evolution, and wrote a great deal on these subjects. He was Fullerian Professor of Physiology at the Royal Institution, and afterwards went to Edinburgh, and finally resided in Oxford, where he died at the early age of forty-six. At Oxford he founded the "Romanes" lecture, which this year was delivered by Professor Weismann.

The ceremony of laying the foundation-stone of the new buildings of the Royal Alexandra Children's Hospital at Rhyl will be made especially interesting, as on that occasion a free cot is to be presented to the hospital as a wedding gift to the Princess May from the county of Shropshire.

Two interesting ceremonies will take place at Poplar on June 11th, when their Royal Highnesses the Prince and Princess of Wales have consented to open the new buildings of the Poplar Hospital for Accidents (which are now completed), and also the Mission for Seamen's Institute, at four o'clock. The Princess will receive purses on the occasion in aid of the institute, the cost of which has not yet been defrayed.

Mr. Lewis H. Glenton-Kerr has been appointed to the office of secretary of the Great Northern Central Hospital. Mr. Glenton-Kerr has occupied the post of assistant secretary at the Middlesex Hospital during several years, where his services have been much appreciated, and the experience he has gained will prove of much value in his new position, on the attainment of which we offer him our congratulations.

His Royal Highness the Duke of Cambridge will make the presentation to Dr. Howship Dickinson of his testimonial on June 18, at noon. The presentation will take place, by permission of the Duke of Westminster, a Vice-President of St. George's Hospital, at Grosvenor House. The testimonial, consisting of a portrait of Dr. Dickinson by the Hon. John Collier, a service of silver plate, and an illuminated address, will be on view.

The new chapel at the Gordon Boys' Home, at Chobham, erected in memory of the late Duke of Clarence, was opened by the Prince of Wales on Monday, May 21st. The chapel was dedicated by the Bishop of Winchester, and afterwards a tablet was unveiled by the Prince of Wales, outside the building, setting forth "that it was dedicated to the Gordon Boys' Home for the uses of the Church of England." Before leaving the Prince of Wales reviewed the boys, and visited the gymnasium, &c.

Last Saturday was Hospital Saturday at Birmingham. The result was a collection of £9,022, a sum slightly in excess of that quoted at the end of the day last year. As the contributions eventually amounted to £11,916 19s. in 1893, we can fairly hope that in 1894 the desired sum of £12,000 will be reached. Not a year has passed in which the fund has not shown an increase in spite of the depression in trade. Since last year the women of Birmingham, as well as the men, have been provided with a beautiful convalescent home, and as the numbers who directly benefit will be larger, it is hoped that the contributions will be larger also. The ladies of Birmingham proved what a genuine interest they take in the fund by their self-sacrificing behaviour in pursuing their collecting with energy in spite of cold weather and a steady rain. Although objecting to the practice of street collections, we cannot but admire so much enthusiasm and self-sacrifice.

